# Long term impact of emotional, social and cognitive intelligence competencies and GMAT on career and life satisfaction and career success

**DOI:** 10.3389/fpsyg.2014.01447

**Published:** 2014-12-16

**Authors:** Emily Amdurer, Richard E. Boyatzis, Argun Saatcioglu, Melvin L. Smith, Scott N. Taylor

**Affiliations:** ^1^Department of Organizational Behavior, Case Western Reserve UniversityCleveland, OH, USA; ^2^YSC LtdNew York, NY, USA; ^3^Department of Educational Leadership and Policy Studies, University of KansasLawrence, KS, USA; ^4^Management Division, Babson CollegeWellesley, MA, USA

**Keywords:** Intelligence, life satisfaction, career satisfaction, Emotional Intelligence, competencies

## Abstract

Career scholars have called for a broader definition of career success by inviting greater exploration of its antecedents. While success in various jobs has been predicted by intelligence and in other studies by competencies, especially in management, long term impact of having intelligence and using competencies has not been examined. Even in collegiate outcome studies, few have examined the longer term impact on graduates' careers or lives. This study assesses the impact of demonstrated emotional, social, and cognitive intelligence competencies assessed at graduation and g measured through GMAT at entry from an MBA program on career and life satisfaction, and career success assessed 5 to 19 years after graduation. Using behavioral measures of competencies (i.e., as assessed by others), we found that emotional intelligence competencies predict career satisfaction and success. Adaptability had a positive impact, but influence had the opposite effect on these career measures and life satisfaction. Life satisfaction was negatively affected by achievement orientation and positively affected by teamwork. Current salary, length of marriage, and being younger at time of graduation positively affect all three measures of life and career satisfaction and career success. GMAT (as a measure of g) predicted life satisfaction and career success to a slight but significant degree in the final model analyzed. Meanwhile, being female and number of children positively affected life satisfaction but cognitive intelligence competencies negatively affected it, and in particular demonstrated systems thinking was negative.

## Introduction

Perhaps more than ever before, the career environment appears to be in constant flux. Even with the key work that emphasized a career model wherein individuals become more self-directed or “protean” (Hall, 2002), few could anticipate the impact technology (e.g., Internet, Google, LinkedIn, etc.) and market instability (e.g., the great recession) would have on individuals' careers. Technological advances have made career boundaries more permeable than ever before. Vast amounts of information are now available to us and global collaborations between individuals are daily, routine occurrences. Changes are so rapid and evolving, that it is doubtful we fully understand the impact they are having on how we characterize the concept of a career.

Amidst this flux, there has been an ongoing call for greater clarity as to the drivers of career success (Gunz and Heslin, 2005; Heslin, 2005). Career success has long been defined by objective outcomes (e.g., pay, promotion, etc.). Yet, these have not always been shown to be what equates with career or life satisfaction (Heslin, 2005). This has led researchers to explore subjective outcomes to define career success (e.g., career satisfaction, life satisfaction, etc.) (Hall, 2002; Greenhaus, 2003; Heslin, 2005). Over the years, the findings that *g* (i.e., general mental ability) is a predictor of performance in jobs and success has steadily gained ground, but the relatively low predicted variance and mixed results of unique variance have raised questions (Nisbett et al., 2012).

At the same time, other career scholars argue that careers are becoming increasingly more interdependent as the need for support from others (i.e., career support and psychosocial support) increases (Higgins et al., 2010). Higgins et al. (2010) showed that, as people receive increasing amounts of support over time, they are more positive about their careers later in life. This key support from others to help navigate through increasing career complexity comes to those who have the interpersonal skills to develop relationships whereby others will want to offer such support. Thus, what inherently emerges from the complexity found in today's career environment, is the importance of individuals' ability to learn via relationships with others, for it is in collaboration with others where we begin to make sense of current circumstances and innovate to succeed in the future. Whether these relationships are dyadic, in teams, or assembled in organizations, we propose that career success will depend more than ever on having the emotional and social skills essential to developing strong, trusting, and resonant relationships with others (Boyatzis and McKee, 2005).

In addition, while effectiveness in various jobs has been predicted by demonstrated emotional and social competencies, especially in management (Boyatzis, 1982, 2009; Spencer and Spencer, 1993; Goleman, 1998; O'Boyle et al., 2011; Joseph et al., 2014; Miao et al., 2014), long term impact of using competencies has not been examined. Competency and job performance theory both predict that people will be more effective in managerial and professional jobs when they use competencies relevant to performance frequently (Boyatzis, 1982). Holistic theories of adult development would, therefore, predict that the use of these same competencies would predict life satisfaction (Schein, 1978).

In programs seeking to develop people for promising careers—whether undergraduate or MBA programs—one of the objectives has always been to prepare people to perform well in their professional roles by helping them develop or enhance management and leadership competencies during their programs. In fact, MBA programs have been shown to increase these competencies (e.g., Boyatzis et al., 2002; Boyatzis and Saatcioglu, 2008). On the other hand, surprisingly, no outcome studies have examined the longer-term impact of key emotional and social competencies on graduates' careers or lives.

Our work is unique in contrast to prior work in that it (1) focuses on outcome assessment of MBA management education, (2) presents a long term, lagged impact study, and (3) explores the impact key emotional and social competencies have on three areas of wellbeing related to careers (i.e., life and career satisfaction and career success) and its relationship to *g*. We draw upon theoretical and empirical work on emotional and social competence, intelligence, career and life satisfaction, and career success to inductively explore the relationship between these constructs. To date, there is no single theory that accounts for the relationship among the constructs in question.

Our study contributes to the literature on careers, competency development, and management education outcome assessment in several ways. First, although growing as a viable stream of research, emotional and social intelligence competence is still relatively new and certainly new to investigation in management education programs (e.g., Jordan et al., 2002; Cote and Miners, 2006; Petrides et al., 2007; Lindebaum, 2009; Cherniss, 2010; Walter et al., 2011). Further, the relationship of these competencies to career constructs is a neglected and highly promising area for research.

This study could help link competency theory with career and adult development theory. Our study examines the impact of emotional, social, and cognitive intelligence competence on important constructs of wellbeing. Specifically, we investigate the impact of demonstrated emotional, social, and cognitive intelligence competencies 5 to 19 years after graduation from an MBA program on career and life satisfaction and career success. We hypothesize that the more emotional, social, and cognitive intelligence competence a person demonstrates, as seen by others at graduation, the greater will be the person's life and career satisfaction and career success years after graduation.

Second, unlike many studies that have relied on self-assessment of emotional intelligence (Carmeli et al., 2009), our study assesses students' behavioral demonstration of competence as rated by other informants. In doing so, we overcome many of the challenges related to student self-assessment (Taylor, 2014). Namely, it has been consistently shown that people are frequently both biased and unreliable when they assess their own abilities (Dunning et al., 2004).

Third, although more of an empirical than theoretical contribution, among the MBA outcome studies that have been conducted, none, to our knowledge, have taken a long term approach or endeavored to look at both career and life satisfaction together.

## Theory and hypotheses

### Emotional, social and cognitive intelligence competencies

Emotional intelligence emerged from research done on emotion and social intelligence (Matthews et al., 2002) and was made popular in the 1990s by several scholars (e.g., Salovey and Mayer, 1990; Goleman, 1995). Emotional intelligence encompasses abilities like emotional self-regulation that are not assessed by IQ tests. Emotional intelligence mingles neocortical and subcortical skills, combining affective and cognitive abilities (Goleman, 2006). Competency and job performance theories claim that to be an effective leader, manager or professional, a person needs to apply knowledge in order to influence people toward desired outcomes (Boyatzis, 1982). These capabilities can be called competencies, which Boyatzis (1982) defined as “the underlying characteristics of a person that lead to or cause effective and outstanding performance” (pp. 20–21). Emotional intelligence competencies are the behavioral level of emotional intelligence (Boyatzis, 2009; Cherniss and Boyatzis, 2013).

Although some scholars claim these are not intelligences but competencies (Ashkanasy and Daus, 2005), others as cited above claim that they are a capability emanating from neural activity that appear at a different level than an internal processing ability. Emotional intelligence and emotional competence are intimately related; one emerges from the other (Goleman, 2006; (Cherniss, 2010). Specifically, emotional intelligence competencies are based on a platform of emotional intelligence wherein emotional intelligence competencies mark a fundamental difference from competencies like technical skills, which rely solely on cognitive, IQ-type abilities based in the Task Positive Network (TPN) predominantly in the neocortex.

In their comprehensive meta-analysis, O'Boyle et al. (2011) showed that although all measures of EI had predictive ability regarding job performance, what they called stream 3 measures had the strongest relationship to performance, not MSCETI which is stream 1 or self-perception measures based on the MSCEIT model which is stream 2. This is consistent with the meta-analysis from Joseph et al. (2014). But stream 3 results may mask even stronger relationships because the variety of measures include self-perception measures, like the EQ-i and behavioral measures, like 360 views from informants or coded video and audio tapes of work samples or simulations. In this study, we chose to focus on the behavioral level of EI and SI, and even include a behavioral level variable for cognitive intelligence. The emotional intelligence (EI), social intelligence (SI) and cognitive intelligence (CI) competencies are the behavioral level of the larger constructs called EI, SI and g.

Emotional intelligence competencies have empirically been shown to cause or predict outstanding leader, manager, or professional performance (Boyatzis, 1982, 2009; Kotter, 1982; Thornton and Byham, 1982; Luthans et al., 1988; Druskat et al., 2005)^1^. Conceptual syntheses have also shown a relationship between emotional intelligence competencies and effectiveness (Campbell et al., 1970; Spencer and Spencer, 1993; Goleman, 1998). Cognitive intelligence competencies have also been shown to be effective in these studies.

Synthesizing this prior work, these competencies appear in three clusters: (1) Cognitive intelligence (CI) competencies, such as systems thinking or pattern recognition; (2) Emotional intelligence (EI) competencies, such as adaptability, emotional self-control, self-confidence, initiative, emotional self-awareness, positive outlook, and achievement orientation; and (3) Social intelligence (SI) competencies, such as empathy, organizational awareness, inspirational leadership, influence, coaching and mentoring, conflict management (i.e., negotiation), and teamwork. Several other cognitive capabilities appear to be “threshold competencies” from the research cited (Boyatzis, 1982), meaning they are needed to be adequate, but more use of them does not lead to effectiveness. Given research to date, such threshold competencies would include: knowledge (technical and functional); deductive reasoning, and quantitative reasoning (Boyatzis, 1982).

Two measures of g will be assessed in this study. One behavioral level measure is composed of the two cognitive intelligence competencies, Systems Thinking and Pattern Recognition. The other is a more traditional measure of g, the GMAT (Detterman and Daniel, 1989).

### Outcome assessment: predicting life and career satisfaction and career success

Although outcome studies have shown that an MBA program can add value to a person' s competencies, the longer term impact of outcomes from their development in such programs (i.e., how students act at the time of graduation) has not been shown (Pascarella and Terenzini, 1991; Mentkowski and Associates, 2000). Many argue that the most relevant outcome from management programs is the amount of money people earn. But salary and bonuses are only one possible measure of success in life and careers and often a short term indicator (Luthans et al., 1988). As outcome measures, salary and bonuses are also contaminated by other factors. For example, wages and bonuses vary tremendously according to the type of industry and country. In addition, those graduates going into careers in non-profit organizations or public sector organizations will generally not be making as much as those in large, for-profit companies.

A more inclusive set of desired outcomes than salary is life and career satisfaction. Life satisfaction refers to a cognitive assessment of one's entire life (Diener et al., 1985). Career satisfaction refers to the satisfaction one receives from internal and external aspects of one's career, including income, advancement and developmental opportunities (Greenhaus et al., 1990). Although subject to progress on one's aspirations and expectations, a sense of how well one is doing in life and work appears a more holistic measure of one's progress than simply assessing one's salary.

Components of career and adult development theories claim that early successes stimulate self-confidence, efficacy and a self-image that enhances goal seeking behavior (Alexander et al., 1990). Using competencies in jobs early in one's career would lead to positive reinforcement (i.e., early “wins”). It is likely that this would alter a person's expectations and strivings. In turn, this would lead to greater striving and, if successful, greater satisfaction.

Competency theory would predict that early career use of EI and SI competencies would result in a person being seen as “good with people.” This may lead to more leadership opportunities and positive feedback (Boyatzis, 2009). In contrast, early career use of CI competencies may result in being seen as a problem solver, analyst or strategic thinker, which in turn could lead to opportunities in staff jobs but not necessarily ones associated with moving up the managerial hierarchy (McClelland, 1985).

EI and SI might lead to greater sense of satisfaction with one's career and progress in terms of personal expectations and social comparison theory (Miao et al., 2014). CI might lead to early career success, but interfere with further advancement because people high on CI might focus more on the analytics of the work than on the people, in a similar way that McClelland and Boyatzis (1982) found that Need for Achievement helped people get promoted to middle level management but was negatively related to further promotion over a 20 follow-up. Satisfaction in one's life, assuming that is a larger sense than career satisfaction, might rely more on EI and SI than CI. In part, the differences may be a result of repeated socialization and rewards for using one's CI and neural TPN which also suppresses the Default Mode Network (DMN) and ability to work well with others (Jack et al., 2012).

Recent research has shown a positive relationship between emotional and social intelligence and psychological wellbeing at work (Carmeli et al., 2009). In their study, Carmeli et al. (2009) found that employees with higher EI reported greater self-esteem, life satisfaction, and self-acceptance. There is also evidence to suggest that having greater emotional management abilities is related to feeling more satisfied with one's career (Lounsbury et al., 2003). Cote et al. (2011) investigated whether a prosocial orientation has an effect on career satisfaction, when mediated and moderated by empathetic accuracy and power. Their findings illustrate that when people have greater prosocial orientations and higher power positions their empathetic accuracy is associated with greater career satisfaction. This result implies that people who are able to accurately infer emotions may be more likely to evaluate their career experiences in a favorable light. Unfortunately, the findings do not show predictive power, only an association, leading the scholars to call for more research to investigate the causal link between ability to understand others and career satisfaction. On the other hand, these recent findings lead us to believe there will be a positive relationship between emotional, social, and cognitive competencies and career and life satisfaction and career success.

This study is, in part, a response to the call for further exploration between EI and wellbeing related outcomes. From the literature just reviewed, it appears that emotional and social intelligence are linked to important life and career outcomes in the workplace. However, what is unknown is whether showing high emotional and social competence upon graduation from an MBA program will later predict greater career and life satisfaction.

Another important measure for graduates of professional programs, like MBAs, is career success. Career success refers to a subjective reaction to one's career experiences (Heslin, 2005). Few enter an MBA with the primary concern to grow, mature, or morally and esthetically develop. Most enter such professional programs because they wish to enter a new career or enhance their success in an existing career. Rode et al. (2008) hypothesized that emotional intelligence for recent graduates should be linked to early career success upon entry into the workplace. They explain that people with greater emotional intelligence show a greater ability to adapt to new environments and build strong bonds with others (Lopes et al., 2003), which should help them acquire necessary support in transitioning to the workplace. This study is encouraging since it looks at how emotional intelligence levels in students predict early career success within 2 years of graduation and entry into the workplace. Unfortunately these findings are not significant. Their results show that in early careers only personality predicted career success, whereas behavioral skills and abilities did not. The authors conclude that it is likely that those with less personality but greater abilities may experience success only later in their careers, once knowledge and skills are developed (Dreher and Bretz, 1991). We extend research in this area by examining how emotional, social, and cognitive competence developed through an MBA program can predict career success throughout people's careers.

**Figure 1:**
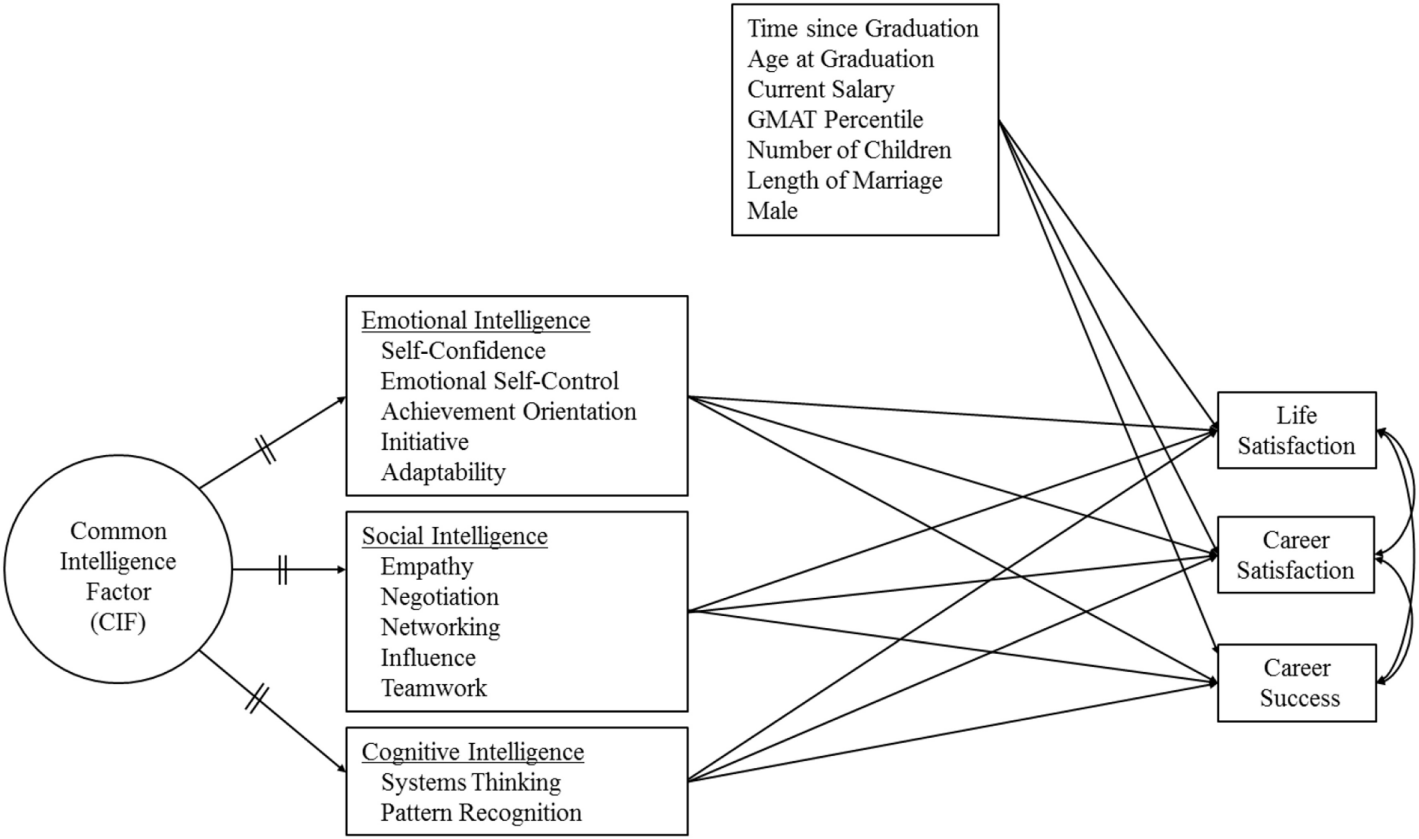
**The model being tested**.

In sum, given the above review of the discussed relationship emotional and social competence have shown to important outcomes, this study was designed to examine the relationship of emotional, social, and cognitive intelligence competence with three dependent career variables, namely life and career satisfaction and career success. Therefore, the model being tested in this study is shown in Figure 1. The specific hypotheses we explore are:

H1. The greater a person's demonstration of emotional intelligence competencies, as seen by others at graduation, the greater the person's life and career satisfaction and career success.
H2. The greater a person's demonstration of social intelligence competencies, as seen by others at graduation, the greater the person's life and career satisfaction and career success.
H3. The greater a person's demonstration of cognitive intelligence competencies, as seen by others at graduation, the greater the person's life and career satisfaction and career success.

## Methods

### Sample and procedure

This study utilizes longitudinal data on the graduates of the MBA program at the Weatherhead School of Management (WSOM), Case Western Reserve University. It focuses on how students' competencies, at the time of graduation, predict their perceived career satisfaction, career success and life satisfaction later on in their work lives. Starting in the early 1990's, data have been collected from all MBA students in the Leadership Assessment and Development (LEAD) course. For a detailed description of the course, see Boyatzis (1994) and Goleman et al. (2002). For past studies examining the impact of the MBA program on the development of emotional, social and cognitive intelligence competencies, see Boyatzis et al. (2002), Boyatzis et al. (1995), Boyatzis et al. (1995) and Boyatzis and Saatcioglu (2008).

We compiled data on the emotional, social and cognitive intelligence competencies from full-time MBA students who graduated between 1992 and 2006. The total number of full-time MBA students graduating between 1992 and 2006 was 1382 (plus the graduates of 1996 and 2003 for whom outcome data was not collected). However, we only had permission to keep data from some of the graduates. Additionally, some of the data was lost over the 15 year span, which resulted in a sample of complete data for 1108 of the graduates.

A number of factors contributed to the smaller number of students' data being available for study. First, letters of informed consent were asked of each student upon completion of the LEAD course. Between 1992 and 2006, the typical permission rate was 85–90%, but in several years of social disarray in the school's administration the permission rate dropped to about 50%, (for further explanation see Boyatzis and Saatcioglu, 2008). When a student did not grant permission, the reasons appeared to be concerns over their privacy, especially for those coming from countries in which such data might be used to harm them, or suspicion about the nature of such outcome research. Second, for early years of 1992 through 1995, randomized sub-samples were used for cost and administrative purposes. For example, the critical incident interview is highly labor intensive for both the researchers and students, so these sub-samples were often 33 to 50% of the graduating group. Third, although attendance at the exit assessment prior to graduation became required in 1998, not all students attending the class completed all of the assessments.

With the sample of 1108, we next matched the graduating data with current addresses from our alumni services department. This narrowed our sample to 975, but we only had graduating data on 625 (46%). Finally, From February 2010 to January 2011, we sent out five email and three direct mail requests for alumni to participate in the present study. The study consisted of one online survey that asked participants to rate their life satisfaction, career satisfaction and career success. Of the letter and email invitations sent, 148 were returned as non-deliverable, which further reduced the potential pool for this study to 477. Graduates had moved, changed companies, countries, or otherwise eluded the donation seeking alumni relations office. In exchange for participating in the study we entered participants in a raffle. We retained a final sample for analysis of 266 of eligible graduates from these cohort years (266/477 = 56%).

Alumni who graduated between the years 1992–1996 made up 21% of the sample. Data from graduates of 1997–1999 and 2002 were accidentally destroyed in two computer crashes and a clerical error of discarding two boxes of data. As such, data from those years do not appear in this paper. Alumni who graduated in 2000, 2001, and 2003 made up 40% of the sample. Finally, alumni who graduated between the years 2004–2006 made up 39% of the sample.

Of this usable sample, 71% were male and the average age was 39. Alumni worked in a number of different industries, such as financial services (20%), manufacturing (14%), consulting services (12%), healthcare (11%), and information technology (9%). The remaining 34% percent were in industries including agriculture, architecture/engineering, construction, education, government, hospitality/tourism, insurance, legal, manufacturing, media services, mining, not-for-profit, publishing, professional science, property management, real estate, transportation, waste management, and retail.

Due to the possible mortality of the sample, we decided to run *t*-tests between the study sample (*n* = 266) and the original entire sample on whom graduating data was available (*n* = 975) with the original sample. We found no statistically significant differences between the two groups on graduating competencies and the demographic and control variables. Therefore, we conclude that the study sample is a reasonably representative sample.

### Dependent variables

*Life satisfaction* was measured with a five item scale (Diener et al., 1985). Sample items included “In most ways my life is close to my ideal” and “If I could live my life over, I would change almost nothing.” Participants rated the extent to which they agreed or disagreed with the statement, using a 7-point Likert scale (1 = strongly disagree to 7 = strongly agree). The scale showed good reliability with α = 0.93.

*Career satisfaction* was measured with Greenhaus et al.'s (1990) five item scale. Sample items were: “I am satisfied with the progress I have made toward meeting my overall career goals” and “I am satisfied with the progress I have made toward meeting my goals for income.” Participants were asked to rate the extent to which they agreed or disagreed with the statement, using a 5-point Likert scale (1 = strongly disagree to 5 = strongly agree). This scale yielded α = 0.94, in this study.

*Career success* was measured with Heslin's (2003) two item scale. The first item asked participants the following, “Everything considered, how successful do you consider your career to date?” Participants used a Likert scale (1= not too successful to 7= very successful). The second item asked participants to rate the level of their success compared to their peers, using Likert responses (1= below average to 7= above average). This scale had good reliability with α = 0.88 in this study.

### Independent variables

The emotional, social, and cognitive intelligence competency assessment items were measured using two different instruments. These instruments included: (1) the Critical Incident Interview (CII), which was a 1-h, audiotaped interview (Flanagan, 1954; Boyatzis, 1982; Spencer and Spencer, 1993) on which the competencies were coded; (2) a 360-degree informant based assessment (i.e., the EAQ and later the ECI-U) (Boyatzis et al., 2002; Boyatzis and Saatcioglu, 2008).

In the CII, coders, who were PhD students, coded 16 competencies in the audiotapes according to the number of times the student displayed each competency. The competencies included: Efficiency Orientation, Planning, Initiative, Attention-to-Detail, Self- Control, Flexibility, Self-Confidence, Empathy, Social Objectivity, Persuasiveness, Networking, Negotiating, Group Management, Developing Others, Systems-Thinking and Pattern Recognition. All of the competencies were coded independently by two or three people and the coders averaged an 89% inter-rater reliability on 16 of the competencies. Because of the cost of doing and coding these interviews, random sub-samples of full time students graduating in the years 1992–1995 were assessed at the time using the CII.

The External Assessment Questionnaire (EAQ) is a 73-item 360-degree format questionnaire in which informants rate the participants on a 1–4 scale on the frequency of which the person being assessed demonstrates each behavior. Informants include a boss, work colleagues, subordinates, professionals, family, fellow students, or friends. There were 21 competencies included in this inventory including: Efficiency Orientation, Planning, Initiative, Attention-to-Detail, Self-Control, Flexibility, Self-Confidence, Empathy, Social Objectivity, Persuasiveness, Networking, Negotiating, Group Management, Developing Others, Systems-Thinking, Pattern Recognition, Oral Communication, Use-of-Concepts, Quantitative Analysis, Use-of-Technology, and Written Communication. The last five items were dropped in 2002, because they did not predict effectiveness in management, leadership or professional jobs (Boyatzis and Saatcioglu, 2008). Students graduating in the years 2000–2004 were rated with the EAQ.

The Emotional Competence Inventory - University (ECI-U) is an expanded version of the EAQ and directly assesses emotional and social intelligence (Wolff, 2007). The ECI-U has the same 360-degree format as the EAQ and asks informants to rate many of the same competencies.

Since three different measures were involved in the outcome assessment studies at different years, we selected only those competencies that had 67% or more of the items the same across measures. That resulted in 12 competencies being analyzed in this study. *Emotional Intelligence* was measured with the five following competencies: Self-Confidence, Emotional Self-Control, Achievement Orientation, Initiative and Adaptability. (α = 0.75). *Social Intelligence* was measured with the five following competencies: Empathy, Negotiating, Networking, Influence and Teamwork (α = 0.82). *Cognitive Intelligence* included the final two competencies of Systems Thinking and Pattern Recognition (α = 0.76).

Since the competencies were measured using three different instruments with different measurement scales (CII, EAQ, ECI-U), we transformed the data by breaking the scores into deciles. We ran frequencies on each of the competencies, and identified the cut-off points for 10 deciles along the percentile distribution. Since each alumnus was only rated with one inventory, we treated the decile scores to represent ability for each competency. For instance, an alumnus who scored in the 70th percentile of displaying influence on the ECI-U, received a score of 7 for the influence competency. As a last step, we created the emotional intelligence, social intelligence and cognitive intelligence clusters, by taking the averages of the decile competencies for each of the respective clusters. For the emotional intelligence cluster we took the average decile scores of self-confidence, emotional self-control, achievement orientation, initiative and adaptability. For the social intelligence cluster we took the average decile scores of empathy, negotiating, networking, influence and teamwork. Finally for the cognitive intelligence cluster we took the average decile scores of systems thinking and pattern recognition.

### Controls

Several variables were specified as controls that prior research has shown to have some impact on either career or life satisfaction, or career success. We included common demographic, work-related controls and general life status controls that may affect satisfaction and success. Specifically we included gender, age at the time of graduation, GMAT percentile, current salary, time since graduation, number of children, and length of marriage.

## Results

Descriptive statistics and two-way correlations for the constructs used in the analysis are shown in Table 1. All correlations among the key measures are in the expected direction. Life satisfaction is correlated only to adaptability (0.13, *p* ≤ 0.05), while career satisfaction is related to achievement orientation (0.16, *p* ≤ 0.05) as well as adaptability (0.17, *p* ≤ 0.01). Career success, on the other hand, is correlated strongly to self-confidence (0.13, *p* ≤ 0.05), achievement orientation (0.14, *p* ≤ 0.05), adaptability (0.16, *p* ≤ 0.01), and networking (0.13, *p* ≤ 0.05). Subsequent analyses test these relationships in a multivariate context, accounting for the effects of several control measures.

**Table 1:** **Means, Standard Deviations and Correlations**.

**Variables**	***M***	***SD***	**1**	**2**	**3**	**4**	**5**	**6**	**7**
1. Life satisfaction	5.30	1.28	*(0.93)*						
2. Career satisfaction	5.43	1.29	0.61^**^	*(0.94)*					
3. Career success	4.90	1.33	0.62^**^	0.73^**^	*(0.88)*				
4. Emotional Intelligence	5.40	1.96	0.06	0.15^*^	0.15^*^	(0.75)			
5. Social Intelligence	5.39	2.12	0.06	0.08	0.09	0.69^**^	(0.82)		
6. Cognitive Intelligence	5.35	2.53	−0.02	0.05	0.06	0.66^**^	0.51^**^	(0.76)	
7. Self Confidence	5.41	2.67	0.02	0.09	0.13^*^	0.71^**^	0.48^**^	0.43^**^	−
8. Emotional Self-Control	5.42	2.78	0.02	0.07	0.04	0.58^**^	0.48^**^	0.34^**^	0.22^**^
9. Achievement Orientation	5.41	2.82	−0.01	0.16^*^	0.14^*^	0.75^**^	0.47^**^	0.53^**^	0.45^**^
10. Initiative	5.41	2.83	0.07	0.07	0.08	0.72^**^	0.43^**^	0.52^**^	0.41^**^
11. Adaptability	5.34	2.75	0.13^*^	0.17^**^	0.16^**^	0.78^**^	0.59^**^	0.51^**^	0.45^**^
12. Empathy	5.37	2.84	0.02	0.10	0.05	0.49^**^	0.77^**^	0.43^**^	0.23^**^
13. Negotiation	5.41	2.70	0.06	0.04	0.02	0.53^**^	0.78^**^	0.43^**^	0.37^**^
14. Networking	5.38	2.83	0.08	0.08	0.13^*^	0.50^**^	0.75^**^	0.30^**^	0.38^**^
15. Influence	5.40	2.82	−0.02	−0.02	0.03	0.53^**^	0.72^**^	0.40^**^	0.48^**^
16. Teamwork	5.38	2.74	0.09	0.10	0.09	0.59^**^	0.79^**^	0.37^**^	0.37^**^
17. Systems Thinking	5.30	2.84	−0.05	0.01	0.01	0.57^**^	0.42^**^	0.90^**^	0.34^**^
18. Pattern Recognition	5.41	2.81	0.01	0.08	0.10	0.61^**^	0.49^**^	0.90^**^	0.44^**^
19. Time Since Graduation	9.73	4.12	0.07	0.10	0.01	−0.12	−0.09	−0.09	−0.12
20. Current Salary	112.25	83.79	0.16^**^	0.29^**^	0.38^**^	0.06	−0.04	0.04	0.10
21. GMAT Percentile	46.78	41.67	0.04	0.03	0.12	−0.02	−0.09	0.09	0.02
22. Number of Children	1.25	1.27	0.28^**^	0.16^**^	0.15^*^	0.12	0.07	0.01	0.04
23. Length of Marriage	7.79	7.10	0.22^**^	0.15^*^	0.12	0.06	0.02	0.00	0.02
24. Age at Graduation	29.55	4.06	−0.12	−0.14^*^	−0.13^*^	0.01	0.05	0.02	−0.09
25. Gender	0.71	0.45	−0.05	−0.02	0.12	0.00	0.02	0.02	0.00
**Variables**	**8**	**9**	**10**	**11**	**12**	**13**	**14**	**15**	**16**
8. Emotional Self-Control	−								
9. Achievement Orientation	0.25^**^	−							
10. Initiative	0.18^**^	0.49^**^	−						
11. Adaptability	0.21^**^	0.46^**^	0.45^**^	−					
12. Empathy	0.50^**^	0.34^**^	0.23^**^	0.44^**^	−				
13. Negotiation	0.31^**^	0.38^**^	0.36^**^	0.44^**^	0.53^**^	−			
14. Networking	0.33^**^	0.32^**^	0.29^**^	0.43^**^	0.41^**^	0.49^**^	−		
15. Influence	0.22^**^	0.36^**^	0.39^**^	0.44^**^	0.43^**^	0.46^**^	0.41^**^	−	
16. Teamwork	0.49^**^	0.39^**^	0.35^**^	0.50^**^	0.54^**^	0.50^**^	0.53^**^	0.44^**^	−
17. Systems Thinking	0.29^**^	0.50^**^	0.44^**^	0.45^**^	0.40^**^	0.35^**^	0.24^**^	0.33^**^	0.29^**^
18. Pattern Recognition	0.32^**^	0.46^**^	0.49^**^	0.46^**^	0.37^**^	0.42^**^	0.29^**^	0.40^**^	0.37^**^
19. Time Since Graduation	−0.12	−0.06	−0.06	−0.07	−0.04	−0.02	−0.10	−0.07	−0.12
20. Current Salary	0.00	0.08	0.04	−0.02	−0.03	−0.02	0.05	−0.10	−0.04
21. GMAT Percentile	−0.04	−0.01	0.00	−0.04	−0.08	−0.02	−0.09	−0.05	−0.12
22. Number of Children	0.10	0.11	0.08	0.09	0.07	0.14^*^	−0.00	−0.02	0.07
23. Length of Marriage	0.07	0.04	0.08	−0.01	0.02	0.09	−0.01	−0.01	−0.01
24. Age at Graduation	0.08	0.08	0.01	−0.06	0.08	0.12	0.02	−0.04	0.02
25. Gender	0.09	0.01	−0.04	−0.05	−0.12	−0.07	−0.02	−0.10	−0.07
**Variables**	**17**	**18**	**19**	**20**	**21**	**22**	**23**	**24**	
17. Time Since Graduation	−								
18. Current Salary	0.61^**^	−							
19. Time Since Graduation	−0.07	−0.10	−						
20. Current Salary	0.04	0.03	0.28^**^	−					
21. GMAT Percentile	0.09	0.08	−0.05	0.10	−				
22. Number of Children	0.07	0.09	0.32^**^	0.24^**^	−0.04	−			
23. Length of Marriage	0.00	0.02	0.47^**^	0.22^**^	−0.05	0.70^**^	−		
24. Age at Graduation	−0.00	0.01	−0.02	−0.08	−0.07	0.16^*^	0.35^**^	−	
25. Gender	−0.02	0.05	−0.03	0.18^**^	0.21^**^	0.18^*^	0.14^*^	0.03	

N ranges from 255-266 for all variables except GMAT Percentile which has an N of 238. Higher numbers represent more Life Satisfaction, Career Satisfaction, Career Success, Emotional Intelligence, Social Intelligence, Cognitive Intelligence, Self-Confidence, Emotional Self-Control, Achievement Orientation, Initiative, Adaptability, Empathy, Negotiating, Networking, Influence, Teamwork, Systems Thinking, Pattern Recognition GMAT Percentile, Number of Children and Age at Graduation. Time since Graduation is 2011 minus the year of graduation and measured in years. Current Salary is rounded to the nearest thousand dollars, 100 = $100,000. Length of Marriage is measured in years. For Gender, 0 = female, 1 = male. Alpha coefficients are on the diagonal in parentheses.

** *p < 0.01*,

* *p < 0.05. Numbers in italics in parentheses on the diagonal are the Cronbach's alpha for these constructed scales*.

Regarding the relationships of EI and SI to g, we find that EI, SI and CI show non- significant correlations with GMAT, which is an approximation of g. We can also see that GMAT shows non-significant first order correlations with each of the dependent variables: life satisfaction, career satisfaction and career success.

We conducted a series of structural equation modeling (SEM) analyses to test the central hypotheses. The general form of the basic structural model is shown in Figure 1. The SEM approach involves two important advantages. First it allows testing effects on several dependent variables simultaneously. As seen in Figure 1, three different dependent variables are predicted by seven covariates and three distinct competence measures, and are also allowed to correlate with one another. The procedure involves three consecutive SEMs. In Model 1, the controls are specified as the only predictors (the baseline model) and GMAT. Model 2 includes emotional, social, and cognitive intelligence competence measures in composite form (no subscales) along with the controls and GMAT. Model 3 is similar to Model 2 but includes the emotional, social, and cognitive intelligence competence subscales rather than the composite measures to examine the independent effects of the specific competencies that collectively constitute the composite competence measures.

The second advantage of the SEM approach is that it includes a latent factor, labeled “Common Intelligence Factor” (CIF) in Figure 1. CIF is specified in Model 2 and Model 3 in order to capture potential overlaps among emotional, social, and cognitive intelligence measures. To model these overlaps, CIF loadings on emotional, social, and cognitive intelligence measures were constrained to be *equal*, depicted by “=” signs on the respective loadings. Model 2 includes only three such equality constraints for CIF loadings, since only three composite competence measures are used. In Model 3, however, a total of 12 equality constraints are specified, one for each of the 12 specific competencies that collectively constitute the composite competence measures. The results of the SEM analyses are presented in Table 2.

**Table 2:** **Results of structural equation models predicting the effects of emotional intelligence, social intelligence, and cognitive intelligence life and career outcomes**.

	**Life satisfaction**	**Career satisfaction**	**Career success**	**Life satisfaction**	**Career satisfaction**	**Career success**	**Life satisfaction**	**Career satisfaction**	**Career success**
**Controls**									
Time Since Graduation	−0.035	−0.025	−0.064^***^	−0.036	−0.017	−0.058^**^	−0.037^*^	−0.020	−0.060^***^
	(0.023)	(0.024)	(0.023)	(0.023)	(0.024)	(0.023)	(0.022)	(0.023)	(0.022)
Current Salary	0.002^**^	0.005^***^	0.005^***^	0.002^*^	0.004^***^	0.005^***^	0.002^*^	0.004^***^	0.005^***^
	(0.001)	(0.001)	(0.001)	(0.001)	(0.001)	(0.001)	(0.001)	(0.001)	(0.001)
GMAT Percentile	0.002	0.001	0.002	0.003	0.001	0.003	0.003^*^	0.001	0.003^*^
	(0.002)	(0.002)	(0.002)	(0.002)	(0.002)	(0.002)	(0.002)	(0.002)	(0.002)
Number of Children	0.247^***^	0.039	0.010	0.247^***^	0.016	−0.010	0.237^***^	−0.012	−0.018
	(0.089)	(0.093)	(0.089)	(0.088)	(0.092)	(0.088)	(0.087)	(0.091)	(0.087)
Length of Marriage	0.034^*^	0.043^**^	0.042^**^	0.033^*^	0.041^**^	0.041^**^	0.035^**^	0.048^***^	0.044^**^
	(0.018)	(0.019)	(0.018)	(0.018)	(0.019)	(0.018)	(0.018)	(0.019)	(0.018)
Age at Graduation	−0.070^***^	−0.066^***^	−0.054^***^	−0.072^***^	−0.062^**^	−0.053^***^	−0.066^***^	−0.067^***^	−0.051^**^
	(0.020)	(0.021)	(0.020)	(0.020)	(0.021)	(0.020)	(0.020)	(0.021)	(0.020)
Male	−0.423^**^	−0.280	0.062	−0.419^**^	−0.282	0.086	−0.445^**^	−0.293	0.096
	(0.182)	(0.190)	(0.182)	(0.182)	(0.188)	(0.181)	(0.178)	(0.187)	(0.180)
**Emotional Intelligence**				0.070	0.161^**^	0.115^*^			
				(0.062)	(0.064)	(0.062)			
Self Confidence							−0.001	−0.001	0.008
							(0.037)	(0.038)	(0.037)
Emotional Self-Control							−0.025	−0.009	−0.050
							(0.034)	(0.035)	(0.034)
Achievement Orientation							−0.061^*^	0.057	0.026
							(0.034)	(0.035)	(0.034)
Initiative							0.055	0.012	0.009
							(0.034)	(0.035)	(0.034)
Adaptability							0.079^**^	0.089^**^	0.097^***^
							(0.036)	(0.038)	(0.036)
**Social Intelligence**				0.019	−0.037	0.024			
				(0.050)	(0.052)	(0.050)			
Empathy							−0.021	0.026	0.030
							(0.036)	(0.037)	(0.036)
Negotiation							0.002	−0.017	−0.024
							(0.038)	(0.039)	(0.038)
Networking							0.033	0.012	0.039
							(0.034)	(0.035)	(0.034)
Influence							−0.071^**^	−0.099^***^	−0.062^*^
							(0.035)	(0.036)	(0.035)
Teamwork							0.069^*^	0.027	0.033
							(0.038)	(0.040)	(0.038)
**Cognitive Intelligence**				−0.085	−0.043	−0.059			
				(0.041)^**^	(0.042)	(0.041)			
Systems Thinking							−0.046	−0.051	−0.067^*^
							(0.036)	(0.037)	(0.036)
Pattern Recognition							−0.025	0.017	0.017
							(0.039)	(0.040)	(0.039)
**Intercept**	7.168^***^	7.000^***^	6.108^***^	7.165^***^	6.424^***^	5.588^***^	7.117^**^	6.704^***^	5.664^***^
	(0.664)	(0.694)	(0.667)	(0.712)	(0.738)	(0.711)	(0.699)	(0.731)	(0.704)
**Correlations**									
Life Satisfection		0.703^***^	0.778^***^		0.688^***^	0.757^***^		0.640^***^	0.703^***^
		(0.100)	(0.100)		(0.098)	(0.097)		(0.091)	(0.091)
Career Satisfection			0.875^***^			0.838^***^			0.772^***^
			(0.106)			(0.103)			(0.096)
**Model fit**									
Chi-Square (d.£)		0.000 (0)			31.572 (22)			298.343 (148)	
p-value					0.085			0.000	
CFI		1.000			0.985			0.894	
TLI		1.000			0.961			0.850	
RMSEA		0.000			0.044			0.068	

*Standardized estimates are reported. Loadings on the common intelligence factor (CIF) constrained to be equal, as shown in Figure 1, are 0.918 (p ≤ 0.010) in Model 2 for EI, SI, and CI, and 1.245 (p ≤ 0.010) in Model 3 for the subscales of intelligence measures. Intercepts for these loadings are not shown*.

* p< 0.100;

** p < 0.05;

*** p < 0.01 (two tailed tests).

The results for Model 1 indicate that people who have higher salaries (β = 0.002, *p* ≤ 0.05), more children (β = 0.247, *p* ≤ 0.01), longer marriages (β = 0.034, *p* ≤ 0.10), are younger at the time of graduation (β = −0.070, *p* ≤ 0.01), and are female (β = −0.43, *p* ≤ 0.05), are more satisfied with their lives. These relationships remain relatively stable across Models 2 and 3, suggesting that the predictors do not interact with the controls in confounding ways. Being further away from graduation appears to have no effect on life satisfaction in Model 1. However, this relationship changes in Model 3, indicating that net of the 12 competencies, being further away from graduation, matters to some degree (β = −0.037, *p* ≤ 0.100).

Regarding g, having stronger GMAT scores does not predict how satisfied people are with their lives in Model 1, but they do in Model 3 (β = 0.003, *p* ≤ 0.050). This is indicative of a likely suppression effect. We address this dynamic further in our later discussion of Model 3.

Graduates with higher salaries (β = 0.005, *p* ≤ 0.010), longer marriages (β = 0.043, *p* ≤ 0.050) and who are younger at the time of graduation (β = −0.066, *p* ≤ 0.010) are more satisfied with their careers. It should be noted that number of children does not significantly predict feeling greater career satisfaction, like it does with life satisfaction. As with life satisfaction, the effects of control measures on career satisfaction remain stable in Models 2 and 3, when the competence measures are introduced.

A similar pattern is observed for career success. Graduates who earned their degrees in more recent years (β = −0.064, *p* ≤ 0.010), those who have higher salaries (β = 0.005, *p* ≤ 0.010) and longer marriages (β = 0.042, *p* ≤ 0.05), and those who were younger at the time of graduation (β = −0.054, *p* ≤ 0.010) feel more successful in their careers. People who have been working for a shorter period of time since graduation also feel more successful, however, as time passes since graduation, sense of achieved success declines (β = −0.064, *p* ≤ 0.010). The control variables again remain stable in Models 2 and 3.

However, like with life satisfaction, GMAT scores become predictive of career success (β = 0.003, *p* ≤ 0.100) only when entered along with competencies in Model 3. This effect is discussed later with the predictor effects in Model 3. Given how stable the control effects are across the models we do not see a pattern of interaction or mediation effects that affect the model.

Model 2 introduces the composite predictors of emotional, social, and cognitive intelligence competence and is significant overall for model fit (chi square= 31.5, *p* < 0.085; CFI= 0.905; TLI= 0.961; RMSEA= 0.044). While having greater emotional competence does not significantly predict how satisfied people feel about their lives, it predicts satisfaction with career (β = 0.161, *p* ≤ 0.050) and career success (β = 0.115, *p* ≤ 0.100). People who have higher cognitive intelligence competence are less likely to be satisfied with their lives (β = −0.085, *p* ≤ 0.050).

Model 3 introduces the 12 individual intelligence competencies as predictors of life satisfaction, career satisfaction and career success. While this models fits the data well (chi square= 298.3, *p* < 0.001; CFI= 0.894; TLI= 0.850; RMSEA= 0.068), a slight drop in fit indices is observed relative to Model 2. This drop plausibly stems from the limited degree of residual dependence among clusters of individual competencies that constitute emotional, social, and cognitive intelligence. When these clusters of competencies are entered as three composite intelligence scores in Model 2 (rather than as 12 distinct measures as in Model 3), residual dependence among them (no matter how small, both within and across clusters) remains a non-issue, resulting in a slightly better overall model fit. The 12 individual intelligence competencies are included in Model 3, in order to provide a broader sense of the drivers of the composite factors. The findings suggest that those with greater achievement drive tend to be less satisfied with their current situation in life (β = −0.061, *p* ≤ 0.100), which is consistent with the expectation that high achievers often strive for greater accomplishments. Adaptability is highly predictive of later life satisfaction (β = 0.079, *p* ≤ 0.050) and career satisfaction (β = 0.089, *p* ≤ 0.05), as well as career success (β = 0.097, *p* ≤ 0.010). It appears that when people are able to accommodate life and career demands, they are better able to appreciate their life and career circumstances.

When it comes to social intelligence competencies, people who use more influence (i.e., which can be viewed as “selling”) are less satisfied with their lives (β = −0.071, *p* ≤ 0.050) and careers (β = −0.099, *p* ≤ 0.010) and view their level of success as inadequate (β = −0.062, *p* ≤ 0.100). As shown here, people with greater teamwork abilities feel more satisfied with their lives (β = 0.069, *p* ≤ 0.100).

The relationship between greater cognitive intelligence competence and feeling less satisfied with life appears to be due to the factor level, since neither systems thinking nor pattern recognition are individually predictive of life satisfaction and thus, the relationship is not being driven by either of the competencies separately. People who show systems thinking in their behavior and communications with others feel less career success (β = −0.067, *p* ≤ 0.050).

Finally, it is important to note that while the other control variables mainly remain stable in Model 3, having a higher GMAT score changes to significantly predict higher life satisfaction and career success. As noted earlier, these changes are likely due to a suppression effect. In Model 1 the suppression is likely due to the omission of the cognitive competencies. This suppression likely reoccurs in Model 2, since cognitive intelligence competence is only introduced in the composite form.

By contrast, in Model 3, when cognitive competence is broken into systems thinking and pattern recognition, systems thinking gains significance with negative career success. It appears to adjust GMAT percentile upward, which suggests that without a behavioral, specific measure of systems thinking, the GMAT percentile effect is slightly biased down. Therefore, people who discuss systems and multiple causal relationships with others might be seen as too analytic by others and hamper their sense of career success. This could suppress the efficacy of their cognitive intelligence “ability” (i.e., how they can think and analyze, but not necessarily explain or discuss the analysis with others as suggested by scoring higher in standardized tests like the GMAT).

As noted earlier, we introduce the competencies in Model 3 in order to see which subscale has the strongest effect independent of the competence composites. However, we believe the higher-order factors to be better predictors of life satisfaction, career satisfaction and career success. We also tested the strength and significance of plausible interaction effects by specifying two-way interactions among the three competency measures, as well as two-way interactions of each competency measure with key control variables, such as time since graduation, GMAT percentile, and age at graduation. None of these interaction effects were large or statistically significant at 0.050 level.

It should be noted that we relied on the “residual centering” approach in constructing our interaction terms to reduce collinearity with main effects which typically results in inflated standard errors for estimated interaction effects. Residual centering is a two-stage procedure that ensures orthogonality between a two-way interaction term and its constituent main effects (see Little et al., 2006). First, the interaction term is regressed (using ordinary least squares procedure) on its two constituent main effects and the residuals from this regression are saved. These residuals are then used to represent the interaction term in the SEM. Relying on residuals in this way isolates the *pure* interaction effect independent of the main effects and thus reduces the potential collinearity that may otherwise inflate the standard error for the interaction term. This procedure is particularly effective in SEM analyses with small data sets, as in our study (Lance, 1988; Marsh et al., 2007).

## Discussion

**Figure 2:**
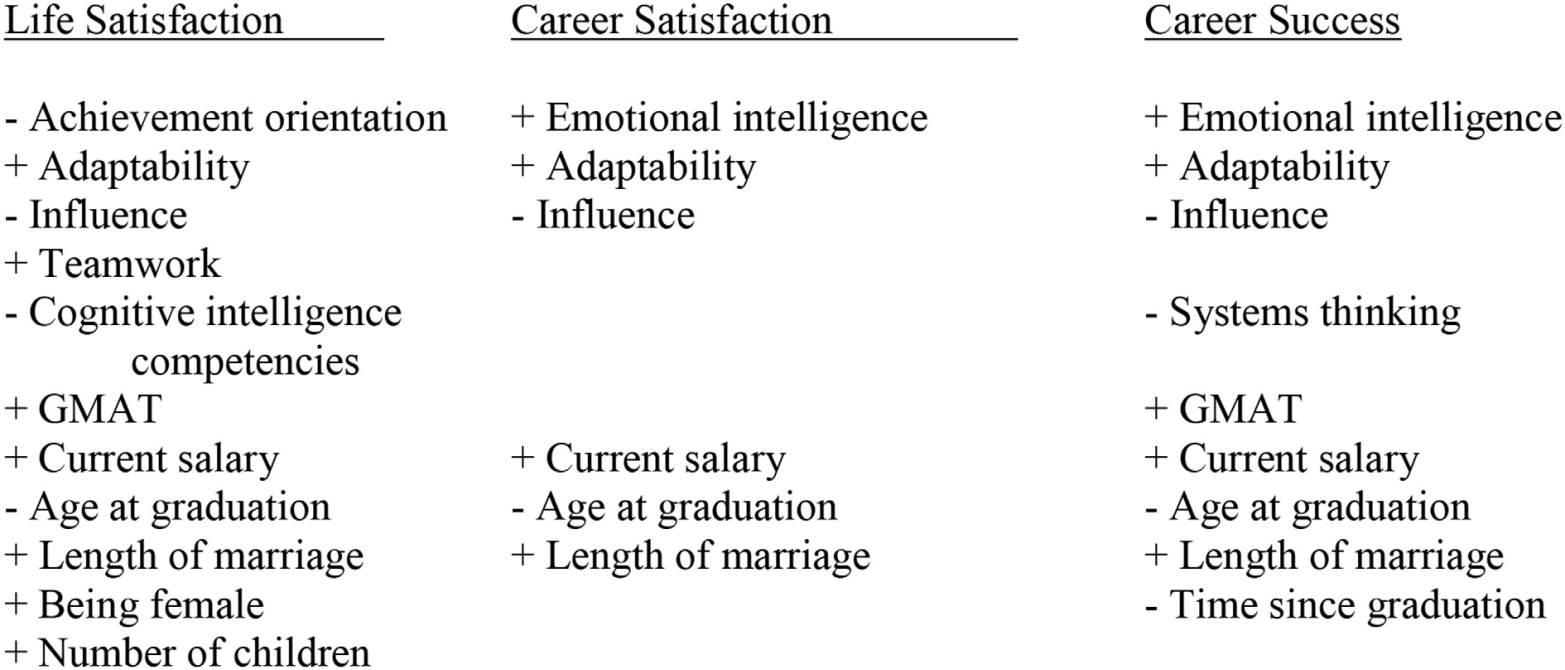
**Summary of significant findings from any of the three models tested**.

The significant findings are summarized in Figure 2. We found that Hypothesis 1 was partially supported, in that demonstrating emotional intelligence competencies at the time of graduation did predict career satisfaction and success, but not life satisfaction. Hypothesis 2 is weakly supported. While SI in composite did not predict life or career satisfaction or career success, when disaggregated into its constituent competencies, teamwork predicted life satisfaction and influence negatively predicted all three of the dependent variables. Hypothesis 3 was slightly supported in a mixed manner. The GMAT measure of g did not show any impact on any of the three dependent variables in models 1 and 2, but did for life satisfaction and career success in Model 3. Furthermore, the cognitive competencies showed a negative effect on life satisfaction in Model 2, and its component of System Thinking showed a negative impact on career success in Model 3.

This confirms the expectations from competency and career theory, and a recent meta-analysis on EI and job satisfaction (Miao et al., 2014). But it does not support holistic adult development theory with a lack of findings on life satisfaction. Our results confirm Lounsbury et al.'s (2003) findings but conflict with Rode et al. (2008) and Carmeli et al. (2009). As suggested by Rode et al. (2008), who did not find support for emotional intelligence and career success, it appears that later career abilities have a positive impact on career success.

With time, employees who display stronger skills and abilities receive greater appreciation which may lead to better opportunities and optimistic career assessments. Carmeli et al. (2009) showed a positive relationship between emotional intelligence and life satisfaction. One possible explanation is that Carmeli et al. (2009) measured emotional intelligence with the Schutte et al. (1998) scale, which is a self-report measure of appraisal, regulation and utilization of emotion. Emotional intelligence in this study was measured by specific competencies and rated by others in addition to the self, but only the others' views were used in this study as a behavioral measure, avoiding the possible self-deception effects of self-assessment (Dunning et al., 2004; Hollander, 2008) and common method bias. Another major difference lies in the design. Carmeli et al. (2009) measured participant's emotional intelligence and then measured career satisfaction 3 weeks later. It is possible that the link between how employees felt about their ability to manage their emotions was due to an overall feeling of general well-being, which accounted for higher ability and satisfaction scores. Our results seem to indicate that over time how well people can manage their emotions as a whole, may not impact how satisfied they are in their lives.

However, in taking a closer look, we see that some emotional competencies do impact assessment of life satisfaction. In particular, demonstrating adaptability positively predicted all three dependent variables and demonstrating achievement orientation negatively predicted life satisfaction. This suggests that people who are emotionally competent at the time of graduation can use some of their emotional competence to cultivate better careers, where they are more satisfied and feel successful. Adapting to life's challenges and taking advantage of opportunities seems to be an important competence to develop in order to respond to the complex world of work and life in 1992—2006. Others have cited adaptability as a “meta-competency” in leadership (Heifetz and Linsky, 2002) and careers (Hall, 1996). It may not be the composite set of competencies that affect life satisfaction as predicted by competency theory, but specific ones. From a holistic adult development perspective, adaptability may be a core capability for all of life's domains.

Adaptability is seen in the evolutionary psychology literature as a core competence (Buller, 2005). It allows a person to adapt or go along with emerging conditions, which is the opposite to the influence competence in which the person seeks to obtain compliance from others and “push” his/her environment. Adaptability also allows a type of problem solving or adjustment. It seems to require noticing and selecting or choosing to which aspect of a situation one should adjust. For example, when a spouse complains about their partner not doing things together, one reaction would be to plan more things. But this ignores the possibility that the real issue is emotional availability and presence with the spouse. Adaptability as a competence allows a person to attend to such changes and when used well provides an ability to adapt more effectively. Adaptability seems less ego-involved in selecting choices as to what to change and probably attaches less self-affirmation to acceptance and continuity to an earlier idea or approach.

As to the negative relationship of achievement orientation and life satisfaction, as we described earlier, McClelland and Boyatzis (1982) showed that achievement motivation helped a person advance from entry to middle-level of management but was negatively related to promotion beyond middle level management. Furthermore, McClelland (1961) explained how people with higher needs for achievement were perpetually dissatisfied—when they reached a goal, they immediately set another one and did not have much interest in relishing or even languishing on the goal attained.

Surprisingly, demonstrating social intelligence competence overall failed to significantly predict any of the dependent variables in the model. These results seem to echo the non-significant findings by Cote et al. (2011) in attempting to predict social understanding on career satisfaction. But this may have been a result of some social intelligence competencies having a positive effect and some having a negative effect, thereby washing out the overall cluster impact. The same dynamic may have contributed to the lack of the emotional intelligence competence cluster showing results for life satisfaction.

Hypothesis 2 was weakly supported in that demonstrating influence had a negative effect on all three dependent variables, and teamwork had a positive effect on life satisfaction. These findings suggest that people who use influence more frequently than others are pushing on their environment and trying to get more from others. This finding may speak to a level of frustration that they feel when others do not respond by showing sufficient impact. The ability to work with others appears to move beyond the team setting and into other aspects of life. When people develop cooperation skills they are more likely to have better relationships in other arenas of life. People who are successful at convincing others may be acting out of a high need to control social interactions. While these people may in fact be successful at controlling social interactions and getting what they need from others, they may subsequently feel dissatisfied when their attempts are unsuccessful. Long term life satisfaction may emerge from less pushing others and more working with others.

A contribution from this study to competency and adult development theory may be that the ability to adapt to events, opportunities and the environment may be more functional in life and careers than asserting oneself on others and trying to change things. In this sense, adaptability, used as just described, satisfies a more Eastern philosophical position.

Other aspects of social intelligence competence have been shown to relate to long term career success, such as networking (Wolff and Moser, 2009), but our findings did not show this effect. The difference might have been that prior work only looked out 3 years and ours was 5–19 years. But this defies common sense and advice for people to network and build and use relationships. It is more likely that the difference between these results and the Wolff and Moser (2009) results is that they used a self-report measure of a person's networks, and we used a behavioral measure of how frequently others saw a person using the networking competence.

Hypothesis 3 was also weakly supported in a complex manner in that demonstrating cognitive intelligence competencies negatively affected life satisfaction, and systems thinking in particular negatively affected career success. But once all variables were entered in to the model, GMAT scores were positively predictive of life satisfaction and career success. This could originate from a dynamic where higher cognitive intelligence competence may stimulate a more skeptical view of life or less social skills and result in others being offended or not “liking” a person at work.

Contrary to more traditional views, higher levels of cognitive intelligence competencies appear to have no bearing on career satisfaction or career success levels. This may be because people come across to others as more abstract, analytic, or intellectual than desired. It may also suggest that people who often see and discuss multiple causal relationships see so many factors in situations that they may imply difficulty in being rational or even display an inability to make a clear or easy decision.

An important distinction between this research and most other comparable studies is that they often use self-assessment measures of a person's competence, EI/SI/CI, or skills. In this study, we used a behavioral measure coming from multiple informants' reports of a person's behavior. The difference has repeatedly been shown as crucial but often research administration convenience overwhelms good sense and practice. For example, McClelland (1985) reported results from a study by Carol Constantian showing a Thematic Apperception Test (TAT) measure of a person's affiliation motives predicted how often a person was with others. In the same study, self-assessed social skills predicted a person's social values, but not their behavior. On the other hand, there may be competencies or arenas of talent that are more sensitive in self-assessment than in demonstrated behavior, such as cognitive competencies involving thought processes.

Another distinction worth noting is that although all of the behavioral competencies assessed in these measures are related to effectiveness in various jobs, most of the studies have been concurrent or 1to 2 year longitudinal studies of performance. In this study, we examined a much longer time period. Even if all of the competencies are predictive of effectiveness in a job, it does not mean they all carry the same weight. The SEM analysis helps us to determine the strongest relationships. In addition, we examined career satisfaction and self-perceived career success, not effectiveness.

Demographic variables showed some interesting effects. Current salary positively predicted life and career satisfaction as well as career success as motivation theory would predict (Herzberg, 1966). Salary is a popular measure or comparative social status indicator of how well one is progressing according to social comparison theory. Being younger at the time of graduation from the MBA predicted all three dependent variables. Relative deprivation theory would claim that older graduates, when adding 5–19 years more, feel that time has slipped by and the future is known rather than a rosy hope (Walker and Smith, 2001). The negative relationship of time since graduation to career success supports this argument that people who had graduated more recently were reporting greater career success.

Length of marriage positively predicted all three dependent variables, but the number of one's children only predicted life satisfaction. Again, Herzberg's theory, which built on Maslow's hierarchy of needs would suggest that salary satisfies needs for security and social status, but a stable marriage and children satisfy higher social needs. Length of marriage suggests stability at home may allow for energy and attention to be devoted at work and to one's career. Having more children may possibly put a strain on people's ability to engage with work. Finally, another interesting result was that being female was positively related to life satisfaction, but gender showed no consistent relationship to the career variables.

Satisfaction is a self-attributed, perception of one's inner state in the context of social comparison. Career satisfaction was distinctly predicted by the set of EI competencies as compared to life satisfaction. But this was most likely because Achievement Orientation negatively predicted life satisfaction. In addition, teamwork, being female and having children led to greater life satisfaction. CI competencies were negatively related to life satisfaction. Achievement orientation and cognitive competencies are more individualistic and analytic. The distinctive positive predictors of life satisfaction are social. These two sets reflect the antagonistic neural domains (Jack et al., 2012). Competency theory explains that behavioral dispositions (i.e., competencies) at graduation increase the likelihood of seeking opportunities for the effective use of these competencies, which in turn reinforce those dispositions. This data suggests the analytic find less social experiences and then may feel they have sacrificed too much in life for work.

### Implications for future career research and for management education practice

People define and redefine their careers over time via learning experiences (Hall, 2002). An opportunity for further research exists in better understanding the relationship between emotional and social competencies and cognitive ability in terms of the sense-making and learning people experience regarding their careers. For example, it seems likely that those who are high in self-awareness and social awareness upon entering the workforce may have a different level of insight in making sense of their career experience than those with lower levels of experience. To better prepare for the complexity of their careers, there has been a call for management educators to help students better understand their emotions to be better equipped to understand the emotions associated with failure and setback during their careers (Kaiser and Kaplan, 2006). We see the work done by career scholars as a key area to draw upon to further scholarly work on emotional and social competence that can in turn influence management education practice. These results also show that more research must be done to explore the different levels of cognitive intelligence, not just fluid and crystalized intelligence and other various forms of general mental ability (Nisbett et al., 2012).

Concern over the efficacy of management degree programs has resulted in outcome assessment becoming standard practice in higher education. Outcome assessment is now required from all management degree programs by the accrediting agencies, such as the Association to Advance Collegiate Schools of Business (AACSB), European Foundation for Management Development, and for universities within the European Union through the Bologna Accord (Porter and McKibbin, 1988). Still, management educators often struggle with how to organize and conduct outcome assessment (Batista et al., 2012). Our study provides an example of how such assessment studies can be conducted.

While we have laid the groundwork for theorizing on the relationship between emotional, social, and cognitive intelligence and subsequent life and career satisfaction, as well as career success, future researchers should continue the development of an overarching theory to explain these relationships. Alternatively, future researchers might further explore the differential impact of specific emotional, social and cognitive intelligence competencies on long-term career outcomes of interest. Developing a greater understanding of how the various competencies impact career and life outcomes could have significant implications for which competencies are targeted for development in management education programs. Doing so will also help working professionals better understand where to target their time and resources to help them navigate turbulent career waters.

In our study, adaptability and teamwork in particular were significant predictors of the selected career outcome measures. While teamwork is often an area of focus for student development, management programs might better prepare their students for successful careers, as well as increase their likelihood of achieving career and life satisfaction by also helping them develop their adaptability. Our results also suggest potential value in helping students become aware of and learn to mitigate the potential negative impact of influence and achievement orientation competencies. While we need to enhance general cognitive ability, it is possible that given the high level of g of people admitted to MBA programs, spending more time attempting to further enhance their fluid intelligence and abstract reasoning may be more of a distraction from other capabilities on which they need development.

This study used a multi-rater assessment to measure student emotional and social competence. MBA students and managers at work are often inundated with self-assessment surveys and the respective feedback from those surveys. On the other hand, as noted earlier, it is well established that people are both biased and unreliable when they assess their own abilities (Dunning et al., 2004), and management education practice and research are not immune to these challenges (Taylor, 2014). Providing students with multi-rater feedback not only serves as a source of outcome assessment upon graduation and beyond (as used in this study), but such feedback will also aid students and managers by proving them more reliable feedback earlier in their careers that can better prepare them for the workplace where the use of multi-rater feedback assessments has become routine (Taylor, 2014).

Finally, our study emphasizes the importance of scholars continuing to investigate the impact of emotional, social, and cognitive intelligence on key outcomes after graduation. This in turn invites management scholars to measure the degree to which management education and leadership development programs are helping graduate students develop emotional and social competence more than the focus on cognitive development. At a time when scholars are making a clarion call for management education to become more relevant (e.g., Druskat et al., 2005; Lorsch, 2009; Bennis, 2010), we believe further study of the development and application of emotional and social competence in management education is an important way to add relevance.

### Limitations

One limitation to this study is the fact that we could not get comparable sized samples from each of the cohort years. While we controlled for time since graduation and age at graduation, the unevenness of the subsamples may have distorted the findings. Another limitation is that we were not able to account for culture or country of origin nor country in which the person has been working. Future research should seek to replicate these results with a sample of professionals who have not been through an MBA program. The degree program itself may bias the original sample. Future research should also examine the pattern of predictive results from self-assessment of the same competencies, as well as the value added of what students learned from their MBA program (i.e., the difference between how they were seen by others at graduation as compared to entry into the program).

Although the compilation of results from three measures might be seen as a limitation, The SEM analysis reported here was replicated for only those respondents whose scores were based on either the EAQ or the ECI instruments which had predominantly the same items (67% of the items were the same, and others were similar). We excluded respondents whose scores were based on the CII which might be considered to overlap with the other two to a lesser degree. The objective of auxiliary analysis was to determine if the estimates reported here were sensitive to potential differences across the three instruments. The results indicated negligible differences compared to findings shown in Table 2 and none that changed levels of significance, suggesting that our estimates were not sensitive to differences across the instruments used for key measures in the study.

Finally, it is important to note that our results are limited in their generalizability given the sample we have used in this study. Participants in this study were not representative of the general population, but instead were MBA graduates and, therefore, likely have an above average cognitive intelligence.

## Conclusion

A person's emotional, social and cognitive competencies can predict life and career satisfaction and career success years, if not decades later. It increases the importance that should be placed on development of these competencies in management and leadership development, whether in a graduate program or organization-based training and development. Our work also extends the call for career scholars to further study the ways in which emotional and social competencies can redefine how we understand careers and career success.

### Conflict of interest statement

The authors declare that the research was conducted in the absence of any commercial or financial relationships that could be construed as a potential conflict of interest.

## Footnotes

^1^See also the special issue of the *Journal of Management Development* in February, 2008 on “Competencies in the 21^st^ Century,”; special issue of the *Journal of Management Development* in April, 2009 on “Competencies in the EU”; special issue of the *Journal of Cross-Cultural Management*, 2012, called “Emotional and Social Intelligence Competencies: Cross Cultural Implications).
